# Community-based infectious disease prevention awareness through children's creative works: outreach embedded in school-based action research

**DOI:** 10.3389/fpubh.2026.1954389

**Published:** 2026-10-28

**Authors:** Asae Oura, Hiroko Yako-Suketomo, Yukiko Naito, Mayu Nakata, Tsugushi Hayashi, Hirofumi Ohnishi

**Affiliations:** 1 Division of Public Health, Department of Social Medicine, Sapporo Medical University School of Medicine, Sapporo, Japan; 2 Department of Sports and Health Science, Faculty of Physical Education, Japan Women's College of Physical Education, Tokyo, Japan; 3 Public Health Laboratory, School of Allied Health Sciences, Kitasato University, Sagamihara, Japan; 4 Department of Psychology, Faculty of Humanities, Sapporo International University, Sapporo, Japan

**Keywords:** action research, creative activities, health education, infectious disease prevention, public health, school health, school-based health promotion

## Abstract

**Background:**

Collaborating with schools, a key social and educational environment for children, is considered beneficial for implementing health education. Although child-focused public health activities are conducted globally, few have been documented in the academic literature.

**Objective:**

This project aimed to provide children with opportunities to engage in infectious disease prevention and health-related activities.

**Methods:**

From 2018 to 2024, we collaborated with schools to conduct public health awareness activities and contests using posters and Senryu created by children in Sapporo. Senryu is a short form of Japanese poetry. This activity was designed as an outreach activity within the framework of action research.

**Practice:**

In the early stages of the project, principals and other school administrators served as the primary contacts for the research team. As the project progressed, however, Yogo-teachers, specially trained school health professionals in Japan who are responsible for health education, first aid, and the coordination of school health activities, became the main collaborators.

**Conclusions:**

We succeeded in sustaining a long-term regional academic partnership. To integrate public health education into school activities, the cooperation of school personnel dedicated to the wellbeing of children was essential. Researchers, together with stakeholders from local governments, companies, organizations, and local communities, cooperated in running the contest. Overall, this project illustrates how sustained collaboration with schools, particularly through the engagement of Yogo-teachers, can facilitate the integration of public health education into routine school practice and support the development of long-term community-academic partnerships.

## 1 Introduction

Public health activities promoted by citizens have contributed to improving the quality of life of the general public in various ways (1, 2). In Japan, there has been growing participation of non-expert youth in community health education programs. One example is the installation of vending machines offering healthy products on university campuses (3). Such health education activities are also consistent with the principles of the Sustainable Development Goals (SDGs) (4). By participating in community activities, young people are expected not only to improve their community but also to increase their social capital with their neighbors; such activities are widely accepted by society as desirable. There have been reports on local health education activities by university students (3). However, there have been very few peer-reviewed reports on such activities by high school students and younger students, especially elementary school students, despite their widespread implementation globally.

Currently, inquiry-based educational activities are being promoted in Japan (5). As children pursue their own interests through inquiry-based learning, they learn about society's structure, human relationships, and social capital. They also develop a sense of responsibility to benefit society based on the experience and skills they have gained. Internationally, student participation has also been recognized as an important component of school health promotion. A systematic review reported that student participation in school health promotion activities may have positive effects on students, school organizations, and social relationships within schools (6). In Japan, child participation is also embedded in school health activities, with teachers supporting children's voluntary participation in these activities (7). Participatory approaches have been applied to health-related learning in Japanese primary schools; for example, the “Creative Health” project incorporated scientific and creative thinking, teamwork, presentation, and opportunities for children to express their own ideas (8). School-based health education has also been applied directly to infectious disease prevention, including a Japanese handwashing education program combining researcher-led instruction with follow-up activities involving teachers and parents (9). In Japan, the current state of inquiry-based learning in education depends on the amount of time each student can spend and how well teachers are prepared to guide them. The Ministry of Education, Culture, Sports, Science and Technology promotes collaboration between schools and local communities, including partnerships with external organizations (10). This school-based and community-linked approach can also be viewed within the health-promoting school's framework, which emphasizes whole-school engagement and collaboration between schools and local communities (11).

In step with a social trend that embraces diversity (12, 13), schools worldwide are fostering education designed to help children with diverse backgrounds live and learn together. In response to this global trend in education, Japan's educational policy is seeking to incorporate the principles of international inclusive education into the domestic system, in alignment with the Convention on the Rights of Persons with Disabilities (14). However, alongside these reform efforts, the number of students in Japan who do not attend school regularly continues to rise annually (15). These statistics suggest that addressing the issue of such students is essential for translating the international principles of inclusive education into practice within Japan. Internationally, systematic school-based approaches to attendance difficulties have emphasized supportive school environments, personalized interventions, and collaboration with families, with some evidence suggesting potential improvement in student attendance and engagement (16). Japan's School Education Act requires guardians to enroll their children in designated types of school (17). There is a growing trend toward establishing in-school support rooms and accommodating children who utilize off-campus day schools (18). Children experiencing difficulties in attending school nevertheless remain members of the school community, and providing opportunities for them to participate, where feasible, in activities shared by other students may provide an important opportunity for educational engagement. Schools are not only places for academic learning but also social environments in which children develop relationships, participate in school life, and build a sense of belonging (19). Educational activities that integrate local social resources, such as those from companies and universities, are being encouraged and promoted (14).

Since 2017, awareness-raising activities have been conducted at schools and children's centers, mainly in Sapporo, to encourage evidence-based behaviors that have been shown to prevent infectious disease (20). As part of these initiatives, we organized an annual poster and Senryu awareness contest for children, ongoing for 7 years. The case study summarizes the outcomes of these long-term community-based educational activities.

## 2 Methods

This manuscript presents a practice-based case study rather than an evaluation of effectiveness. This public health research project was designed as action research that began in 2017, in Sapporo City, Japan (Figure 1). The public health activities began in 2017 at one children's center and were subsequently expanded to multiple children's centers and schools from 2018 onward. The results of the action research in this study have been reported previously (20–23). This initiative was planned as an outreach activity based on the principles of action research (24, 25). Participating schools and facilities were defined as those that agreed to take ownership of the initiative and incorporate it into their educational activities or organized events. Only works submitted as part of these institution-led activities were included in the counts reported in this study. Participation in the outreach activities did not necessarily entail entry into the contest. Accordingly, participation in the outreach activities without contest entry was not included in the reported counts. Exceptional participation outside this framework was also permitted. For example, some students who had transferred to other schools wished to continue participation and submitted works directly to the university, without researcher involvement at their new schools. Siblings who were not part of the designated participant group occasionally joined events at participating facilities. Their works were accepted as exceptional submissions but were excluded from the reported counts. This approach was based on the action research principle of prioritizing the needs and interests of participating schools and facilities, with efforts made to accommodate requests to participate wherever possible. Works submitted through these exceptional forms of participation were excluded from this report to ensure that reported counts reflected activities conducted within the participating schools and facilities. Because schools and facilities coordinated the collection of submissions, they informed the research team which works were outside the scope of the reports. The basic policy of this activity was to “prioritize the interests of the field.” The goal of the activity was set as “to encourage children to wash their hands more than they do now,” so that all adults involved could understand it. Children selected one prevention method they understood, illustrated it and displayed it in a public location. As the project progressed, we recognized that creating posters required art materials and considerable time, which limited the settings in which children could participate. We therefore considered it necessary to provide a simple submission category that children could engage in using their own ideas without requiring art materials. Accordingly, Senryu was introduced as an additional submission category in the 2019 school year. Senryu is a short Japanese poetic form traditionally composed in a 5-7-5 sound-unit pattern and often used to express observations of everyday life. In addition, a learning partner model (26, 27) was intentionally designed so the information could be disseminated to family and friends. The Japanese school year runs from April to March. Awards ceremonies for each school year's entries were held early in the following school year. The 2019 awards ceremony, which had been scheduled for June 2020, was suddenly canceled because of the COVID-19 pandemic.

**Figure 1:**
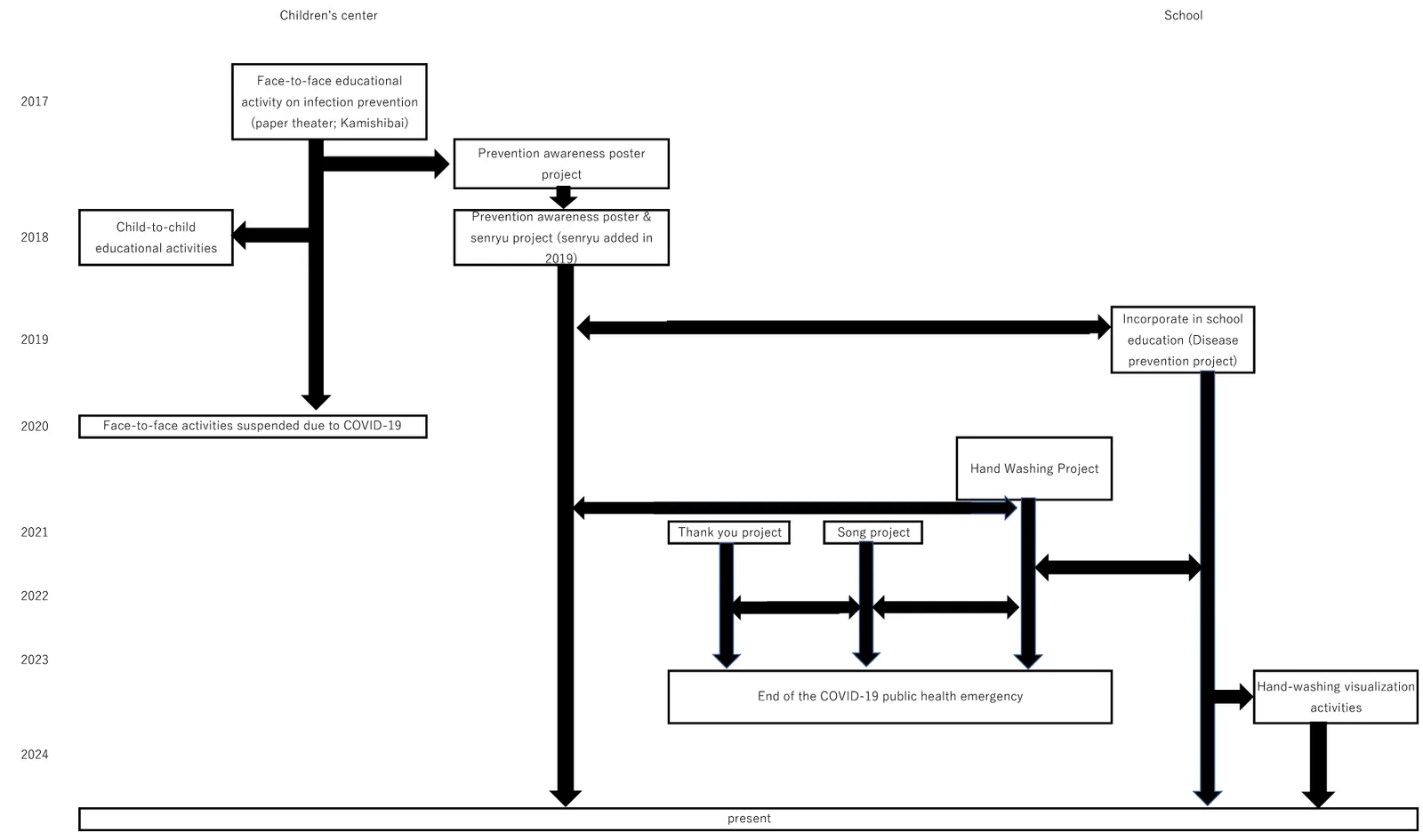
The role of prevention awareness posters and senryu activities within the infectious disease prevention education and research project.

Focusing on the children's activities, the active period generally runs from the end of summer vacation through the autumn and winter months, when awareness of infectious disease prevention was typically heightened. The deadline is set after the winter break in January, and the winners are announced in early May. In other words, the collection of all artworks was generally completed around February each year. At schools participating in the action research, this outreach activity was conducted concurrently with the lessons themselves. To submit an entry, children were required to select an infection prevention method they understood and create either a poster or Senryu by the deadline. Entries were collected by participating schools and then submitted to the university. The submission guidelines included explanations regarding permission for the works to be used in public health activities and disclosure of contributors' names if their works received an award. In the context of action research, the PDCA cycle was structured as follows (28): The “Plan” phase spans the period from the conclusion of the previous year's collection of entries until the awards ceremony. During this time, the project team confirms the schools' intent to participate, refines the plan, and shares it with stakeholders. The “Do” phase covers the active period following the summer vacation through the completion of the selection process and the awards ceremony. This is the time dedicated to the actual creation of entries at the schools. Schools that complete their entries immediately transition to an internal promotional phase, while regional public relations activities commence after the submission deadline. The “Check” phase takes place during the month following the awards ceremony. Each school analyzes what went well, what did not, and any unexpected events to identify the underlying causes. When possible, proposals for improvement are shared with stakeholders. These insights gained are incorporated into the following year's activities as “Action (A).”

The judges were selected through direct negotiations with the authors. They selected individuals and companies that shared the research philosophy and were willing to collaborate without compensation. Companies were contacted directly through their customer service departments by telephone or email. To promote fairness, the entries were judged anonymously. Apart from this procedure, the evaluation of the works was left to the discretion of each judge. The selection process for works is illustrated in Figure 2.

**Figure 2:**
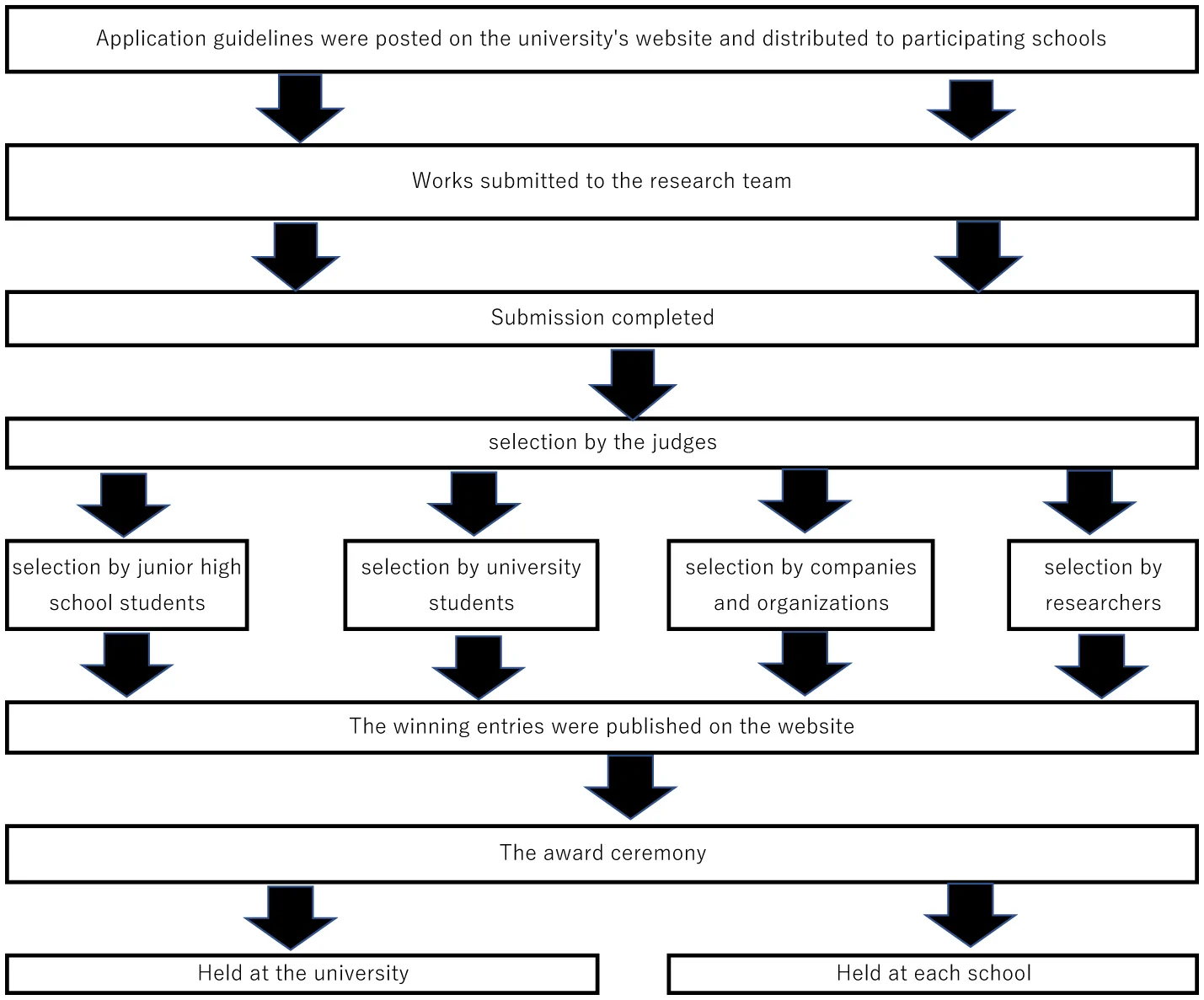
The process from submission of works to the award ceremony.

In this report, the researchers compiled and organized the utilization and outcomes for each year based on records of the submitted entries and relevant policies. Microsoft Excel was used for this compilation. From the 2020 school year onward, some participants submitted multiple works, and some schools handled the matching of submissions to individual contributors internally. Some submissions were anonymized before being sent to the research team, and identifying individual contributors would have required further inquiry with the schools. Because the research team could not confirm the exact number of unique participants, we reported the number of submitted works throughout the study period. The final count of submitted works was verified. No formal qualitative academic analysis was conducted. Winning works from 2020 onwards are available online (29–33). While the 2019 works were not published online, the information was distributed to the winners. Other evidence includes submitted works, newspaper articles, television broadcasts, photographs and videos, lists of people in charge, portfolios on the websites (34), and research notes. Comments conveyed to the researchers by school and facility staff were used to describe reported secondary effects. Reports of secondary effects conveyed by school and facility staff were shared among the researchers when they were received. As noted in the Limitations section, no direct evidence, such as email or audio recordings, remained. This evidence is based solely on hearsay. This study took every possible measure to prevent individuals from being identified. Only comments that the researchers could identify and confirm, or for which multiple similar testimonies were obtained, were used as supporting evidence. The criterion for adopting comments was defined as “outcomes not originally anticipated by the research team during the preparatory stage.” A final review of comments provided by schools and facility staff regarding the reported secondary effects was completed. The action research, including the research use of outcomes obtained from these educational activities, was reviewed and approved by the Ethics Committee of Sapporo Medical University. The study was initially approved in fiscal year 2017 (approval no. 29-2-34), prior to the commencement of the study in 2018. As the action research developed, subsequent amendments to the research plan were reviewed and approved, and the current approval number is 1-2-22. The outreach activities were conducted as educational activities or organized events under the responsibility of participating schools and facilities. The schools and facilities determined how the activities were implemented and provided information about the activities to children and their guardians in advance. Participation was managed according to the usual procedures of each school or facility; therefore, the specific methods used to provide information and confirm participation varied among institutions and were not uniformly verbal or written. The research team did not directly recruit individual children or their guardians or obtain separate written informed consent for research participation from them. The research team subsequently used the outcomes of these school- and facility-led educational activities for research purposes in accordance with the research protocol approved by the Ethics Committee. The outreach activities were conducted with pre-planned procedures in place for handling requests from individuals who wished to not participate or to not have their names disclosed. To date, no such requests have been received. Since participation in the educational activities was managed by the schools and facilities, the research team did not collect any information other than the student's name, school name, and grade level.

## 3 Summary of practice

Table 1 shows annual trends in schools participating in outreach activities and designated personnel. In the early stages, the contact person was often the school administrator. However, after the activities became widely known, Yogo-teachers—specially trained school health professionals in Japan who specialize in school health and health education—frequently assumed this role. This increase was partly attributable to the transfer of participating schools from our other activity, the “Hand-washing Project.”

**Table 1:** Annual trends in schools participating in outreach activities and designated personnel.

	2018	2019		2020		2021		2022		2023		2024		Liaison officer's position		
	Poster	Poster	Senryu	Poster	Senryu	Poster	Senryu	Poster	Senryu	Poster	Senryu	Poster	Senryu	management	Yogo-teacher	others
Junior high school																
A	74													✓		
B				1	47	1	65							✓		
C				6	15	6	11		15						✓	✓
D						31		12	9	23	48	21	21		✓	
E							22		10		12	1	26		✓	
F							534		515		457		244		✓	
G													201			✓
Elementary school																
A	12	27	13	2	4	11	11	5		9	300		138	✓		
B		43		162		103	1	29		71				✓		
C	164	19		47		39		26						✓		
D				155	193	55	89							✓	✓	
E					92			3		5	84	4	131			✓
F					69											✓
G							1	7			4			✓	✓	
H								1	99		48			✓		
I									178					✓		
J										7					✓	
K										7	26	7	3		✓	
L												22	42	✓		
M												24	35		✓	
N													55		✓	
Children's center																
A	3													✓		
B	45	27	41											✓		
C	34													✓		
D	101	28	23											✓		
E	17	5												✓		
F	24													✓		
G	41													✓		
H	23	20	26							10				✓		
I	9													✓		
J	12													✓		
K	11	7	7											✓		
L		4	14											✓		
M		17	12											✓		
N		65	42											✓		
O		15												✓		
P		23												✓		
Q		4												✓		
R		16	26											✓		
Kindergarten																
A				7						11				✓		
Total number	570	320	204	380	420	246	734	83	826	143	979	79	896			

As shown in Table 1, overall submissions increased from 2019 to 2021, a period spanning the onset of COVID-19. Table 2 summarizes the public outreach activities conducted during the study period. This period also coincided with increased media coverage and public visibility of the project. In 2020, although face-to-face outreach activities were suspended due to the COVID-19 pandemic, the project was adapted to methods that could be implemented safely, and children's creative works were disseminated through media-based channels. We also added new activities as much as possible and continued the activities from the previous year. In some cases, a single participant submitted multiple works.

**Table 2:** Summary of public outreach activities.

	2018	2019	2020	2021	2022	2023	2024
Expected activity							
Conducting activities as part of school class		✓	✓	✓	✓	✓	✓
Use the work in health activities at schools			✓	✓	✓	✓	✓
Utilizing the information for school-based health activities in the local community	✓	✓	✓	✓	✓	✓	✓
Exhibition outside the facility		✓	✓	✓	✓	✓	✓
Exhibition at subway stations or community centers		✓	✓				✓
Exhibition at City Hall				✓	✓	✓	✓
Public health videos made by children were presented on a publicly installed screen in the underground mall			✓	✓			
Other activities (children-to-children about hand-washing lesson, quizzes, etc.)		✓	✓	✓	✓	✓	✓
Media coverage and public dissemination							
Featured in local magazine	✓	✓	✓				
City newsletter							
School-run website				✓	✓	✓	✓
Newspaper publication		✓	✓	✓	✓	✓	✓
TV coverage	✓		✓		✓		

Figure 3 shows the PDCA cycle for incorporating new outreach activities. Regarding collaboration with stakeholders, approval was obtained from schools, organizations, and government agencies, after which a person in charge was appointed, and a formal handover process was conducted when personnel changed.

**Figure 3:**
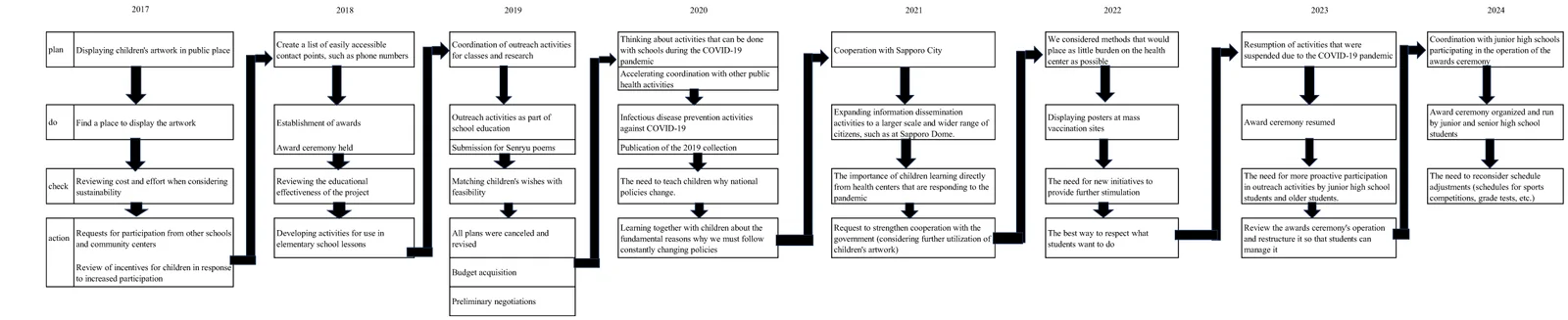
PDCA cycle in outreach activities.

Starting in 2021, some schools began to show enthusiasm for spreading children's activities. Collaboration also expanded beyond prevention and awareness to encompass other school-based initiatives.

Although the 2019 awards ceremony, which was scheduled for June 2020, was canceled because of the pandemic, junior high school students had participated in the selection of the 2019 entries as judges for the first time (35). To acknowledge their dedicated efforts and contributions, the researchers devised a plan to incorporate the students' selection process and critiques into the collection of works. Additionally, amidst the pandemic, some schools began incorporating Senryu writing into homework assignments for junior high school students.

Although not shown in the table, we identified some secondary benefits of these activities through comments from schoolteachers and staff at the children's center. The following are anecdotal observations. One case involved a child who attended school but was unable to enter the classroom. Encouraged by the Yogo-teachers to create an entry, the child spent considerable time on the project. According to the schoolteacher, the child was overjoyed upon receiving an award. The schoolteacher also reported that the child subsequently returned to the classroom. At other schools, this activity included over 20 hours of inquiry-based learning. Before the children submitted their work, we held an in-school competition to share what other children had been working on. Some children who were unable to attend school reportedly participated and received awards. According to the teacher, one student reportedly said at the time, “I am so happy that everyone voted for me.” The schoolteacher also reported that the student later resumed attending school. Additionally, staff at a children's center reported that one child, who had exhibited behavioral difficulties and had frequently been reprimanded by teachers, received an award. They also reported that the child later took the initiative to instruct younger students on proper hand-washing. These anecdotal observations describe outcomes reported by educational staff during the project and may provide hypotheses for future studies.

## 4 Discussion

A previous report suggested that developing what children can do is beneficial in addressing developmental challenges (20). The sustained implementation of this activity may have been supported by various organizational efforts, the involvement of collaborators, and the children's participation.

We also believe that this activity serves as an example of the application of Kolb's experiential learning model (36), which is used in medical education. Specifically, we intentionally incorporated a process that encouraged reflection. Anecdotal reports from teachers and facility staff suggested that participation may have been associated with increased self-confidence in some students. These reported outcomes may reflect not only participation in the contest itself but also education and guidance provided by teachers and facility staff. UNESCO has emphasized the importance of preparing teachers to meet children's diverse learning needs, particularly those of disadvantaged learners (37).

At the start of the project, the research team only expected students to create artwork following simple participation rules. However, in reality, schoolteachers integrated the activity into their educational programs in ways that reflected the specific circumstances of local schools, resulting in diverse implementation strategies. In this process, unexpected results arose, such as students creating multiple pieces of artwork beyond the initial rule of “one piece per student.” Rather than strictly applying the initial protocol, the research team adjusted the project based on on-the-ground experience, modified the rules as needed, and used the insights gained from each cycle to improve subsequent implementations. The project evolved through this collaborative and adaptive process, consistent with the iterative and participatory nature of action research (38).

The COVID-19 pandemic substantially changed the broader context of infectious disease prevention and public health communication (39). The increase in submissions and project visibility observed during this period should therefore be interpreted cautiously.

Over time, a group of schools that remained highly engaged in the project emerged, and the scope of research activities expanded. A particular feature was the increase in the number of Yogo-teachers involved in the project. Even in schools where the principal was the main leader, a Yogo-teacher often served as a bridge between the students and researchers, supplementing knowledge and listening to students' motivations and requests to further develop the activity. Thus, the role of Yogo-teachers in this project extended beyond serving as contact persons for the research team and included broader involvement in the development of school-based health promotion activities. This is consistent with the established role of Yogo-teachers in Japanese school health, which includes health education and a broad range of school health activities (40). From a broader perspective, the health-promoting school's framework emphasizes a holistic approach as well as engagement and collaboration between schools and local communities (11).

The researchers also encouraged participation in the competition at schools other than the cooperating school through newspapers and websites, but the number of participants from schools that positioned the activity as part of their education program was overwhelmingly higher. Over time, sustained collaboration among participating schools, researchers, and other stakeholders appeared to develop into more established working relationships. This process may be viewed in light of Tuckman's classic model of group development, which describes how groups develop over time toward more established roles and task-oriented functioning (41).

The issues to be addressed in future action research are as follows. First, securing evidence is the greatest challenge that must be addressed. Typically, teachers prioritize protecting children's privacy and avoid communicating via email, fax, or other means of leaving their records. Consequently, similar measures tend to be taken even for positive comments that could be made public, resulting in casual conversations. Second, we must continue to ensure the freedom of work selection. This is because when rules are unified, works that demonstrate greater techniques or are created in accordance with certain rules are more likely to be selected. To preserve children's creativity, it is essential that they think independently, create their own work, and feel confident that their efforts will be accepted.

The following points describe the applicability of the study. First, it is crucial for teachers, who are liaisons with schools, to be interested in these activities. In fact, in our project, a retired teacher who served as a local school supporter assisted the activities at the children's center for several years and eventually introduced the activities to a school. Second, researchers should actively seek out collaborators from outside their fields and professions who share common ideals. Having individuals with diverse perspectives judge the works contributes to greater diversity.

The continuity of these activities is described below. The ultimate goal is to make this a self-sustaining activity in the local community, independent of researchers. A trial was conducted during the 2024 award ceremony (34). Junior and senior high school students were responsible for all aspects of the event, including venue setup, reception, hosting, photography, and cleanup.

The limitations of this study are as follows. First, this activity was conducted during the unique period of the COVID-19 pandemic. As people's awareness of infection prevention is much higher than usual, caution is required when extrapolating this finding to other activities. Second, the collaboration with the Hand-washing Promotion Project may have resulted in an overestimation of the level of participation. The hand-washing promotion project provided free foam soap for use in schools as a reward for students participating in each public health activity. Therefore, it is possible that teachers recruited more students than usual to obtain soap. Third, there may have been a bias in which students' positive comments were actively shared with the researchers, but their negative comments were not. In addition, because this project was conducted within the Japanese school system, including collaboration with Yogo-teachers, its applicability to educational settings in other countries should be considered with caution.

This is a report on prevention awareness posters and Senryu promotional activities conducted by children in Sapporo from 2018 to 2024. We successfully sustained a long-term regional academic partnership that integrated public health education into school-based activities. The cooperation of schoolteachers—whose commitment to children's wellbeing enabled the continuity and depth of these initiatives—was essential for embedding public health learning within comprehensive educational practice.

## Data Availability

The datasets presented in this article are not readily available because the materials underlying this report are the posters and senryu created by the participants. The only personal information collected by the research team was students' names, school names, and grade levels. Requests to access these materials will be considered in accordance with the scope of the ethics approval and applicable privacy requirements. Any materials shared in response to such requests will have students' names, school names, and grade levels removed. Requests to access the datasets should be directed to Asae Oura, aoura@sapmed.ac.jp.

## References

[1] World Health Organization. Health Promotion. Available online at: https://www.who.int/health-topics/health-promotion (Accessed September 9, 2026).

[2] Kuroda A Murayama H Kurotani K Fukuda Y Kuwahara K. A descriptive study on volunteer activities of providing meals during the early phase of the COVID-19 pandemic: clarifying the activities process and preliminary investigation of the effect of activities on residents. Nihon Koshu Eisei Zasshi. (2022) 69:284–96. doi: 10.11236/jph.21-097 35228469

[3] Kagawa Nutrition University. Healthy and Sustainable Campus Project: Logo Finalized, Beverage Replacement, and Information Provision Launched (2024). Available online at: https://www.eiyo.ac.jp/news/2024/0528info.html (Accessed September 9, 2026).

[4] United Nations. The 17 Goals (2015). Available online at: https://sdgs.un.org/goals (Accessed September 9, 2026).

[5] Ministry Ministry of Education Culture Sports Science and Technology. Period for Integrated Study and Inquiry. Available online at: https://www.mext.go.jp/a_menu/shotou/sougou/main14_a2.htm (Accessed September 9, 2026).

[6] Griebler U Rojatz D Simovska V Forster R. Effects of student participation in school health promotion: a systematic review. Health Promot Int. (2017) 32:195–206. doi: 10.1093/heapro/dat090 24395957

[7] Tomokawa S Miyake K Takeuchi R Kokudo S Asakura T. Participation of children in school health in Japan. Pediatr Int. (2020) 62:1332–8. doi: 10.1111/ped.14347 32542791

[8] Goto A Lloyd Williams A Okabe S Koyama Y Koriyama C Murakami M . Empowering children as agents of change to foster resilience in community: implementing “creative health” in primary schools after the Fukushima nuclear disaster. Int J Environ Res Public Health. (2022) 19:3417. doi: 10.3390/ijerph19063417 PMC8955969 35329108

[9] Ueno M Miyake K Shimada H Tomokawa S. Effectiveness of a continuous handwashing education program with multiple activities at a Japanese kindergarten school. Health Promot Int. (2024) 39:daae056. doi: 10.1093/heapro/daae056 38864399

[10] Ministry Ministry of Education Culture Sports Science and Technology. Case Studies for Promoting Collaboration Between Schools and Local Communities. Available online at: https://manabi-mirai.mext.go.jp/jirei/chiiki-jirei/index.html (Accessed September 9, 2026).

[11] World Health Organization United Nations Educational Scientific and Cultural Organization. Making Every School a Health-Promoting School: Global Standards and Indicators. Geneva: World Health Organization and UNESCO (2021).

[12] UNESCO. The Salamanca Statement and Framework for Action on Special Needs Education (1994). Available online at: https://unesdoc.unesco.org/ark:/48223/pf0000118118?locale=en (Accessed September 9, 2026).

[13] United Nations. Convention on the Rights of Persons with Disabilities (2006). Available online at: https://www.un.org/development/desa/disabilities/convention-on-the-rights-of-persons-with-disabilities.html (Accessed September 9, 2026).

[14] Central Council for Education. Reiwa no Nihon-gata Gakko Kyoiku [The Japanese Model of School Education in the Reiwa Era]: Final Report (2021). Available online at: https://www.mext.go.jp/content/20210126-mxt_syoto02-000012321_2-4.pdf (Accessed September 9, 2026).

[15] Ministry Ministry of Education Culture Sports Science and Technology. Survey on Problem Behaviors and School Refusal among Students (2024). Available online at: https://www.mext.go.jp/content/20241031-mxt_jidou02-100002753_2_2.pdf (Accessed September 9, 2026).

[16] Boaler R Bond C. Systemic school-based approaches for supporting students with attendance difficulties: a systematic literature review. Educ Psychol Pract. (2023) 39:439–56. doi: 10.1080/02667363.2023.2233084

[17] Ministry Ministry of Education Culture Sports Science and Technology. School Enrollment in Elementary and Lower Secondary Schools. Available online at: https://www.mext.go.jp/a_menu/shotou/shugaku/ (Accessed September 12, 2026).

[18] Ministry Ministry of Education Culture Sports Science and Technology. Support Measures for School Refusal Students; COCOLO Plan (2023). Available online at: https://www.mext.go.jp/content/20230418-mxt_jidou02-000028870-cc.pdf (Accessed September 9, 2026).

[19] Stremfel U Sterman Ivancic K Peras I. Addressing the sense of school belonging among all students? A systematic literature review. Eur J Investig Health Psychol Educ. (2024) 14:2901–17. doi: 10.3390/ejihpe14110190 39590027 PMC11592540

[20] Oura A Ohnishi H Nagata Y Hasegawa J Terada Y Suketomo H . Public health activities to prevent infectious disease in collaboration with children's center. Hokkaido Koshu Eiseigaku Zasshi. (2019) 33:51–3.

[21] Oura A Ohnishi H Koyama M Nagata Y Hasegawa J Suketomo H . Practical challenge of elementary school for infection prevention: the period of integrated study for school children. JJHEP. (2021) 29:215–22. doi: 10.11260/kenkokyoiku.29.215

[22] Oura A Ohnishi H Koyama M Nagata Y Hasegawa J Suketomo H. . The journey to launching “Let's protect children in our community! Operation to defeat COVID-19.” *Hokkaido no Koushu Eisei*. (2021) 47:65–6.

[23] Oura A Naito Y Yako-Suketomo H Nakata K Koyama M Ohnishi H. Science behind children's handwashing: action study of 9- to 10-years-old elementary school students in Japan. Front Public Health. (2024) 12:1425646. doi: 10.3389/fpubh.2024.1425646 39091534 PMC11293302

[24] Smith L Rosenzweig L Schmidt M. Best practices in the reporting of participatory action research: embracing both the forest and the trees. Couns Psychol. (2010) 38:1115–38. doi: 10.1177/0011000010376416

[25] Japanese Society of Health Education and Promotion. Guidelines for Writing an Action Research Paper. Available online at: https://nkkg.eiyo.ac.jp/_src/11109/action%20reserch%20guideline%20final.pdf?v=1665457314355 (Accessed September 9, 2026).

[26] Navarro AM Raman R McNicholas LJ Loza O. Diffusion of cancer education information through a Latino community health advisor program. Prev Med. (2007) 45:135–8. doi: 10.1016/j.ypmed.2007.05.017 17604831 PMC2043119

[27] Yako-Suketomo H Oura A Katayama K Saito K Ohashi K Navarro AM. The effect of a learning partner model-based program on spreading cancer prevention knowledge using community health volunteers in Japan. J Prim Care Commun Health. (2022) 13:21501319221110682. doi: 10.1177/21501319221110682 PMC9274416 35801585

[28] Coughlan P Coghlan D. Action research for operations management. Int J Oper Prod Manag. (2002) 22:220–40. doi: 10.1108/01443570210417515

[29] Sapporo Medical University. COVID-19 Infection Prevention Awareness Posters and Senryu: 2020 Award-Winning Collection (2021). Available online at: https://web.sapmed.ac.jp/jp/news/topics/jmjbbn000000ysvm.html (Accessed September 9, 2026).

[30] Sapporo Medical University. COVID-19 Infection Prevention Awareness Posters and Senryu: 2021 Award-Winning Collection (2022). Available online at: https://web.sapmed.ac.jp/jp/news/topics/idhlv70000001wfr-att/idhlv70000001wlm.pdf (Accessed September 9, 2026).

[31] Sapporo Medical University. COVID-19 Infection Prevention Awareness Posters and Senryu: 2022 Collection (2023). Available online at: https://web.sapmed.ac.jp/jp/npm4n200000012xv-att/bvrbcc0000002bcv.pdf (Accessed September 9, 2026).

[32] Sapporo Medical University. COVID-19 Infection Prevention Awareness Posters and Senryu: 2023 collection (2024). Available online at: https://web.sapmed.ac.jp/jp/sotca300000016hb-att/tqirmb0000002cwy.pdf (Accessed September 9, 2026).

[33] Sapporo Medical University. COVID-19 Infection Prevention Awareness Posters and Senryu: 2024 Collection (2025). Available online at: https://web.sapmed.ac.jp/jp/t18h550000000n8g-att/t18h550000000ztx.pdf (Accessed September 9, 2026).

[34] Sapporo Medical University. Handwashing Promotion Project and Public Health Activities, and Research Visualizing Handwashing Among Students and Children (2024). Available online at: https://web.sapmed.ac.jp/jp/news/topics/nvs2110000000sxc.html (Accessed September 9, 2026).

[35] Sapporo Medical University. Infectious Disease Prevention Education for Junior and Senior High School Students (2020). Available online at: https://web.sapmed.ac.jp/jp/news/photo/jmjbbn000000otx3.html (Accessed September 9, 2026).

[36] Kolb DA. Experiential Learning: Experience as the Source of Learning and Development. Englewood Cliffs, NJ: Prentice Hall (1984).

[37] UNESCO. Teaching and Learning: Achieving Quality for All; EFA Global Monitoring Report 2013/4. Paris: UNESCO (2014). Available online at: https://unesdoc.unesco.org/ark:/48223/pf0000225660 (Accessed September 9, 2026).

[38] Baum F MacDougall C Smith D. Participatory action research. J Epidemiol Community Health. (2006) 60:854–7. doi: 10.1136/jech.2004.028662 PMC2566051 16973531

[39] World Health Organization. WHO COVID-19 Policy Brief: Building Trust Through Risk Communication and Community Engagement (2022). Available online at: https://apps.who.int/iris/handle/10665/362670 (Accessed September 9, 2026).

[40] Tomokawa S Miyake K Asakura T. Sustainable human resource training system for promoting school health in Japan. Pediatr Int. (2020) 62:891–8. doi: 10.1111/ped.14292 32374493

[41] Tuckman BW. Developmental sequence in small groups. Psychol Bull. (1965) 63:384–99. doi: 10.1037/h0022100 14314073

